# The Management of Persistent or Recurrent Variceal Bleeding After Injection Sclerotherapy by Somatostatin

**DOI:** 10.1155/1992/86987

**Published:** 1992

**Authors:** S. A. Jenkins, R. Shields, N. Jaser, S. Ellenbogen, E. Naylor, J. N. Baxter

**Affiliations:** University Department of Surgery 5th Floor, U.C.D. Royal Liverpool University Hospital Prescot Street Liverpool L69 3BX UK

## Abstract

Sixteen patients with persistent (*n* = 11) or recurrent (*n* = 5) variceal bleeding after injection sclerotherapy and balloon tamponade were treated with an intravenous infusion of somatostatin 250μg/ h. Somatostatin infusion successfully controlled the bleeding in 15 of the 16 patients but one rebled after 72 h of treatment. In one patient with poor liver function (Child’s C) bleeding was not controlled by somatostatin, further injection sclerotherapy or balloon tamponade of the oesophagus. The results of this study, although uncontrolled and with a small number of patients, suggest that somatostatin is a very effective treatment for the control of post-injection sclerotherapy variceal bleeding.


*HPB Surgery*, 1992, Vol. 5, pp. 221-227
Reprints available directly from the publisher
Photocopying permitted by license only

© 1992 Harwood Academic Publishers GmbH
Printed in the United Kingdom

### THE MANAGEMENT OF PERSISTENT OR RECURRENT VARICEAL BLEEDING AFTER INJECTION SCLEROTHERAPY BY SOMATOSTATIN

S.A. JENKINS, R. SHIELDS, N. JASER, S. ELLENBOGEN, E. NAYLOR and J.N. BAXTER

*University Department of Surgery, Royal Liverpool University Hospital, Prescot Street, Liverpool, UK*

*(Received 25 September 1991)*

Sixteen patients with persistent ($n$ = 11) or recurrent ($n$ = 5) variceal bleeding after injection sclerotherapy and balloon tamponade were treated with an intravenous infusion of somatostatin 250μg/h. Somatostatin infusion successfully controlled the bleeding in 15 of the 16 patients but one rebled after 72 h of treatment. In one patient with poor liver function (Child's C) bleeding was not controlled by somatostatin, further injection sclerotherapy or balloon tamponade of the oesophagus. The results of this study, although uncontrolled and with a small number of patients, suggest that somatostatin is a very effective treatment for the control of post-injection sclerotherapy variceal bleeding.

KEY WORDS: Oesophageal varices, haemorrhage, injection sclerotherapy, balloon tamponade, somatostatin

### INTRODUCTION

Injection sclerotherapy is successful in controlling acute variceal bleeding in 85-95% of patients[1,2]. If bleeding persists immediately after completion of the sclerotherapy or recurs early (within 24 h), haemostasis can be achieved in the majority of cases by balloon tamponade of the oesophagus. However, tamponade of the oesophagus is associated with a complication rate of approximately 25%[3] and the risk of perforation or ulceration may be increased after injection sclerotherapy since the sclerosant renders the mucosa more friable[4]. Consequently, it has been recommended that mechanical compression of the oesophagus should not exceed 12 h after sclerotherapy[3]. Therefore if the patient continues to bleed or rebleeding occurs early after balloon tamponade instituted to control haemorrhage after injection sclerotherapy, there is a need for an alternative therapy to achieve haemostasis. In view of the reported efficacy of somatostatin in the control of acute variceal bleeding[5-10], we describe our experience with the hormone in controlling significant bleeding from the varices after failed injection sclerotherapy and balloon tamponade.

Address correspondence to: S.A. Jenkins, University Department of Surgery, 5th Floor, U.C.D., Royal Liverpool University Hospital, Prescot Street, Liverpool L69 3BX, UK

### PATIENTS AND METHODS

Between January 1985 and December 1988, 106 patients presented with acute variceal haemorrhage which was treated with injection sclerotherapy.

Injection sclerotherapy was always carried out under general anaesthesia using a William's overtube. Every attempt was made to inject 2-3ml of ethanolamine oleate intravariceally. On some occasions however, the sclerosant was accidentally injected paravariceally. If significant oozing persisted after two 10 min periods of compression with the overtube, balloon tamponade of the oesophagus was instituted for 12 h (Minnesota tube, gastric balloon 300 ml air, oesophageal balloon 40 mm Hg).

During the study period 11 patients continued to bleed or experienced early recurrent variceal haemorrhage ($n = 5$) within 12 h after a period of balloon tamponade instituted to achieve haemostasis after injection sclerotherapy. In all these patients, the continued or recurrent variceal bleeding was significant as defined by either (1). a systemic disturbance (blood pressure less than 100 mm Hg and pulse rate greater than 100 beats min) requiring blood transfusion to maintain the vital signs or (2). the necessity to transfuse two or more units of blood in 24 h to maintain the haemoglobin levels.

The causes of portal hypertension, grading of hepatic function according to the criteria of Child[11], age, sex, blood transfusion requirements and the duration of somatostatin therapy are shown in Table 1.

**Table 1** Details of patients with persistent or recurrent varieceal haemorrhage after injection sclerotherapy

| | |
|---|---|
| Number of patients (M/F) | 16 (11/5) |
| Age Median (range) | 56.5 (39–73) |
| Child's grading | |
| A | 3 |
| B | 5 |
| C | 8 |
| AETIOLOGY: | |
| Non-cirrhotic | |
| Extrahepatic block | 2 |
| Cirrhosis | |
| Alcoholic | 10 |
| Cryptogenic | 3 |
| Primary biliary cirrhosis | 1 |
| Blood transfusion requirements (units) | |
| After Injection Sclerotherapy prior to Somatostatin infusion | |
| Median (range) | 5 (3–12) |
| After commencement of somatostatin infusion | |
| Median (range) | 3 (2–11) |
| Period of somatostatin treatment (h) | |
| Mean Median (range) | 72 (48–120) |

### *Somatostatin Administration*

All 16 patients received a continuous infusion of 250 μg/h somatostatin. Special precuations were taken to ensure that the somatostatin infusion was not interrupted even for a short period, e.g. overlapping of the infusion bags. As soon as the infusion was established all patients received a bolus injection of 250 μg somatostatin which was repeated daily for the duration of the treatment. If for any reason the infusion was interrupted, a further bolus dose of 250 μg was administered as soon as it was re-established.

### *Success of Treatment*

Successful control of haemorrhage was defined as the cessation of bleeding as evidenced by the absence of overt signs or by a stable haematocrit and haemoglobin levels without need for blood transfusion.

### RESULTS

During the study period 33 patients (31%) required balloon tamponade of the oesophagus immediately after injection sclerotherapy to control persistent oozing from the varices despite two periods of compression with the overtube. Bleeding was controlled in 17 patients (19%) by 12 h of balloon tamponade. Since we consider balloon tamponade of the oesophagus to control persistent oozing from the varices after injection sclerotherapy an integral part of the procedure, the overall success rate of injection sclerotherapy was 90/106 (85%).

Bleeding persisted or recurred (within 12 h) from the varices in 16 patients (15%). A continuous infusion of somatostatin controlled bleeding in 15 of the 16 patients (94%). No further bleeding occurred in 14 patients and 12 were placed on an elective injection sclerotherapy programme to obliterate their varices (Figure 1). Two patients died without any recurrent haemorrhage during their period of hospitalisation, one from hepatic failure and the other from a myocardial infarction (Figure 1). One patient rebled after 72 h treatment with somatostatin and underwent a second course of injection sclerotherapy. The bleeding persisted despite a further period of balloon tamponade and somatostatin infusion. An emergency oesophageal transection was carried out which controlled the bleeding and this patient made an uneventful recovery (Figure 1).

Bleeding was not controlled by somatostatin in one patient and persisted despite concomitant balloon tamponade and further injection sclerotherapy (Figure 1). An emergency oesophageal transection controlled the bleeding but the patient died 20 days post-operatively from hepatic and renal failure.

### DISCUSSION

Injection sclerotherapy is a very effective treatment for controlling bleeding oesophageal varices, haemorrhage being controlled in approximately 85-90% of patients[1,2]. Occasionally bleeding persists after a course of injection sclerotherapy, either emergency or elective, and it is the policy in our unit to use balloon tamponade immediately after the procedure if massive oozing continues. However, balloon tamponade of the oesophagus is not without side-effects, among which oesophageal perforation and necrosis resulting in ulcer formation are among the more serious. Furthermore, the risks of these two complications may be increased after injection sclerotherapy because of the deleterious effects of the sclerosant on

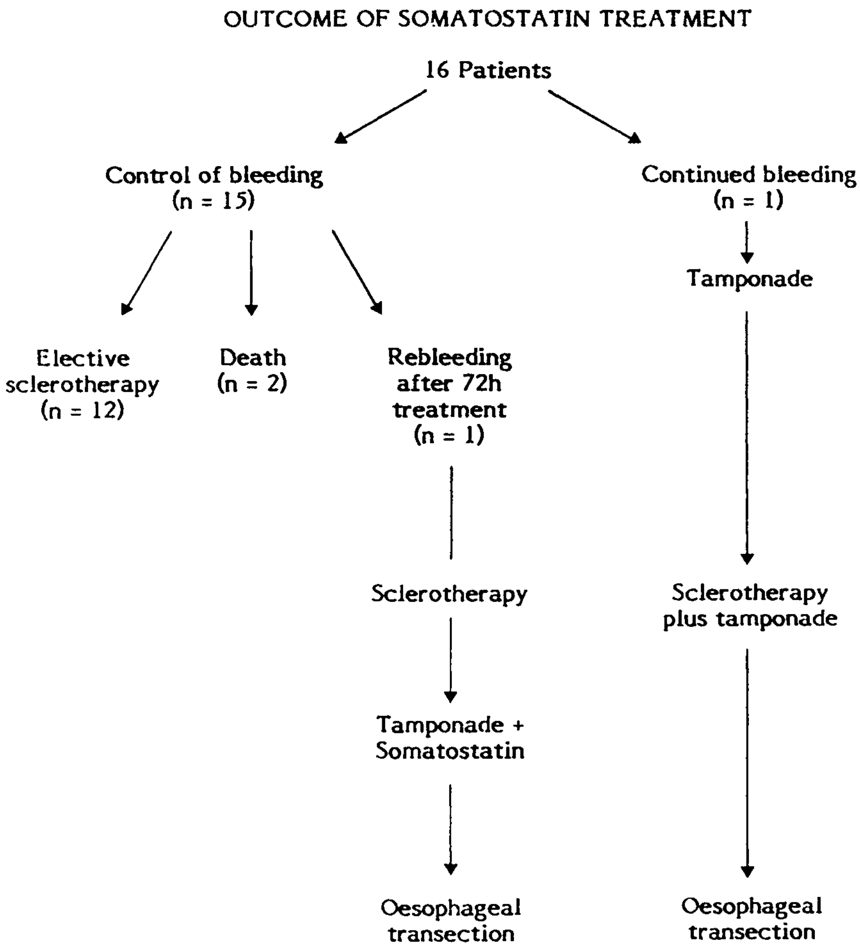

**Figure 1** Outcome of patients receiving somatostatin after the failure of injection sclerotherapy and 12 h balloon tamponade to control bleeding.

the oesophageal mucosa[4]. Consequently, it has been suggested that the period of balloon tamponade of the oesophagus be restricted to 12 h to minimise oesophageal perforation and ulceration and other deleterious side effects[3]. If bleeding continues after 12 h of tamponade or recurs early after removal of the tube control of the haemorrhage can be problematical. Further periods of balloon tamponade of the oesophagus may control bleeding, but the complication rate may be unacceptably high. Similarly, a second course of injection sclerotherapy may control the haemorrhage, but in those patients in which bleeding is not controlled the mortality approaches 100%[2]. Furthermore, emergency oesophageal transection with abdominal devascularisation is often unrewarding because of the difficulties in mobilising the oesophagus after previous injection sclerotherapy[11] and the high morbidity and mortality of emergency surgical intervention to control bleeding in patients with poor liver function[12-17].

The results of the present study clearly indicate that somatostatin infusion is a very safe and effective treatment for the control of substantial variceal haemorrhage after failed injection sclerotherapy followed by 12 h balloon tamponade. Thus initial control of bleeding was achieved in 15 of the 16 patients without any undesirable side effects. The patient who rebled after 72 h of treatment with somatostatin (Child's B) had moderate liver dysfunction and bleeding was controlled by oesophageal transection from which she made an uneventful recovery. The one patient in which somatostatin failed to control the bleeding had severe liver dysfunction (Child's C) with large varices and haemostasis was not achieved by further injection sclerotherapy and balloon tamponade. Although bleeding was controlled by emergency oesophageal transection the patient died post-operatively from progressive hepato-renal failure, the usual outcome of emergency surgery in patients with severe hepatic dysfunction. Therefore, overall somatostatin was effective in controlling post injection sclerotherapy in 87.5% of the patients.

The precise mechanism whereby somatostatin is effective in controlling variceal haemorrhage has not been fully established. Somatostatin has been demonstrated to reduce portal pressure in both experimental animals and in patients with cirrhosis[18-20]. However, perhaps more importantly, the hormone has a much greater effect on collateral and azygous blood flow than on portal pressure[21-22]. Consequently, a reduction in collateral blood flow, including that through the varices may be a major factor in controlling hamorrhage. Furthermore, although erosion of the varices by reflux of gastric content is not thought to contribute to variceal bleeding *per se*, the marked inhibitory effect of sandostatin on basal and stimulated gastric secretion of acid and pepsin[22-24] may present clot dissolution and hence recurrent haemorrhage.

In summary therefore, somatostatin would appear to be a safe and effective therapy for the control of variceal bleeding after injection sclerotherapy. Clearly, however, further randomised controlled trials are necessary to substantiate this hypothesis. Furthermore, since the risk of rebleeding is greatest in the first 5 days after variceal haemorrhage[23,24], somatostatin or its synthetic analogue somatostatin, may be a useful routine adjuvant therapy to injection sclerotherapy to reduce the risk of recurrent haemorrhage. Again, however, further studies are required to substantiate this hypothesis.

*Acknowledgements*

This study was supported by grants from the Mersey Regional Health Authority and Serono Laboratories Ltd.

*References*

1. Terblanche, J., Kahn, D., Campbell, A.H. *et al.* (1983) Failure of repeated sclerotherapy to improve long-term survival after oesophageal bleeding. *Lancet*, **2**, 1328
2. Terblanche, J., Burroughts, A.K. and Hobbs, K.E.F. (1989) Controversies in the management of bleeding oesophageal varices. *New Engl.J.Med.*, **320**, 1393–1398
3. Blavianos, P., Gimson, A.E.S., Westaby, D., *et al.* (1989) Balloon tamponade in variceal bleeding: use and misuse. *Br.Med.J.*, **298**, 1158
4. Evans, D.M.D., Jones, D.B., Cleary, B.K. *et al.* (1982) Esophageal varices treated by sclerotherapy: a histopathologic study. *Gut*, **23**, 615–620

5. Kravetz, D., Bosch, J., Teres, J. *et al.* (1984) Comparison of intravenous somatostatin and vasopressin infusion in the treatment of acute variceal haemorrhage. *Hepatology*, **4**, 442–446
6. Jenkins, S.A., Baxter, J.N., Corbet, W.A. *et al.* (1985) A prospective randomised controlled clinical trial comparing somatostatin and vasopressin in the treatment of acute variceal haemorrhage. *Br.Med.J>*, **290**, 275–278
7. Testoni, P.A., Masci, E., Passareti, E. *et al.* (1986) Comparison of somatostatin and cimetidine in the treatment of acute bleeding oesophageal varices. *Curr.Ther.Res.*, **39**, 758–766
8. Valenzuela, J.E., Schubert, T., Fogel, M.R. *et al.* (1989) A multicentre randomised double-blind trial of somatostatin in the management of acute haemorrhage from oesophageal varices. *Hepatology*, **10**, 958–961
9. Burroughs, D.K., McCormick, P.A., Hughes, M.D. *et al.* (1990) Randomised double blind controlled study of somatostatin for control of variceal bleeding. *Gastroenterology*, **89**, 1388–1395
10. Jenkins, S.A., Baxter, J.N., Ellenbogen, S. *et al.* (1990) A prospective randomised controlled trial comparing somatostatin and injection sclerotherapy in the control of acute variceal haemorrhage. *Br.J.Surg.*, **70**, A702
11. Johnston, A.G. (1982) Oesophageal transection and devascularisation procedures. In. Westaby, D., MacDougall, B.R.D. and Williams, R. Eds. *Variceal Haemorrhage*, pp. 97–103 London: Pitman
12. Wexler, M.J. (1980) Treatment of bleeding oesophageal varices by transabdominal transection with the EEA stapling instrument. *Surgery*, **88**, 406–416
13. Kunzak, L.I. (1981) Use of an EEA stapler in the transection of the oesophagus in severe haemorrhage from oesophageal varices. *Am.J.Surg.*, **141**, 387–390
14. Wannermaker, S.R., Cooperman, M. and Carey, L.C. (1983) Use of the EEA stapling instrument for control of bleeding oesophageal varices. *Surgery*, **94**, 620–626
15. Mir, J., Ponce, J., Morena, E. *et al.* (1982) Esophageal transection and para-oesophagogstric devascularisation performed as an emergency measure for uncontrollable bleeding. *Surg.Gynaec.Obstet.*, **155**, 868–872
16. Durtschi, M.B., Carrico, C.J. and Johansen, K.H. (1986) Esophageal transection fails to salvage high risk cirrhotic patients with variceal haemorrhage. *Am.J.Surg.*, **150**, 18–23
17. Jenkins, S.A. and Shields, R. (1988) Variceal haemorrhage after failed injection sclerotherapy: the role of emergency oesophageal transection. *Br.J.Surg.*, **76**, 49–51
18. Jenkins, S.A., Devitt, P., Day, D.W. *et al.* (1986) The effects of somatostatin on hepatic haemodynamics in the cirrhotic rat. *Digestion*, **33**, 126–134
19. Bosch, J., Kravetz, D. and Rodes, J. (1981) Effects of somatostatin on hepatic and systemic haemodynamics in patients with liver disease: comparison with vasopressin. *Gastroenterology*, **80**, 518–525
20. Jenkins, S.A., Baxter, J.N., Snowdon, S. *et al.* (1988) Effects of somatostatin on hepatic and systemic haemodynamics in patients with cirrhosis and portal hypertension. *Fibrinolysis*, **2**, 48–50
21. Jenkins, S.A., Yates, J., Nott, D.M. *et al.* (1989) The effects of vasoactive drugs in the collateral circulation of rats with portal hypertension. *Br.J.Surg.*, **76**, 635
22. Bosch, J., Kravetz, D., Mastai, R. *et al.* (1983) Azygous blood flow in cirrhosis. Effects of balloon tamponade, vasopressin, somatostatin and propranolol. *Hepatology*, **3**, 855
23. Bloom, S.R., Mortimer, C.H, Thorner, M.O. *et al.* (1974) Inhibition of gastrin and gastric secretion by growth hormone release inhibiting hormone. *Lancet*, **ii**, 1106–1109
24. Raptis, S., Dollinger, H.C., von Berger *et al.* (1975) Effects of somatostatin on gastric secretion and gastric release in man. *Digestion*, **13**, 15–26
25. Gomez-Pan, A., Albinus, M., Reed, J. *et al.* (1975) Direct inhibition of gastric acid and pepsin secretion by growth hormone release inhibiting hormone in cats. *Lancet*, **1**, 888–890

*(Accepted by S.Bengmark 15 November 1991)*

### INVITED COMMENTARY

Injection sclerotherapy is widely accepted as the treatment of choice for acute variceal bleeding. Control of bleeding is achieved in about ninety per cent of patients with a mortality of around 20 per cent, half of which is due to liver failure. Should bleeding recur after the first injection, mose centres are prepared to have a second attempt at sclerotherapy. The use of tamponade in 31 per cent of patients undergoing acute sclerotherapy in this series is very high. Tamponade, although useful, is uncomfortable, risky and temporary. An intravenous injection of 10 mg of metaclopramide controls post-injection oozing in the majority of patients. When two injections fail to control bleeding, it is suggested that emergency transection should be performed since the mortality is high in patients who require three or more injections[1]. However the results of emergency transection after failed sclerotherapy are far from encouraging[2]. Unfortunately the patients who fail to stop bleeding are often in Child's Class C where the mortality for emergency surgery varies from 70-100 per cent.

In many publications, somatostatin and its longer-acting analogue, octreotide (Sandostatin), have been shown to be as effective or better than placebo, vasopressin, tamponade and acute sclerotherapy respectively in the early management of acute variceal bleeding. In this paper the extension of the use of these drugs to the management of post-injection bleeding has produced encouraging results. In the Liverpool series only four patients per annum fall into the category of post injection bleeders, making a controlled trial impossible. Although only half of their patients were Child's Class C patients, the results are encouraging to surgeons reluctant to undertake operative salvage of these difficult patients. Elsewhere the authors have shown that somatostatin is effective whether post-injection bleeding is due to recurrent variceal bleeding, post-injection ulceration or oesophagitis. While waiting for further results from other centres, many of us have been encouraged to adopt octreotide (Sandostatin) therapy for these post-injection bleeders.

### *References*

1. Burroughs, A.K., Hamilton, G., Phillips, A., Mezzanotte, G., Mcintyre, N. and Hobbs, K.E.F. (1989) A comparison of sclerotherapy with staple transection of the esophagus for the emergency control of bleeding from oesophageal varices. *New England Journal of Medicine*, **321**, 857–862
2. Jenkins, S.A. and Shields, R. (1988) Variceal haemorrhage after failed injection sclerotherapy: The role of emergency oesophageal transection. *British Journal of Surgery*, **76**, 49–51

Georg Johnston
Belfast

